# Kaiso Represses the Cell Cycle Gene *cyclin D1* via Sequence-Specific and Methyl-CpG-Dependent Mechanisms

**DOI:** 10.1371/journal.pone.0050398

**Published:** 2012-11-30

**Authors:** Nickett S. Donaldson, Christina C. Pierre, Michelle I. Anstey, Shaiya C. Robinson, Sonali M. Weerawardane, Juliet M. Daniel

**Affiliations:** Department of Biology, McMaster University, Hamilton, Ontario, Canada; Université Paris-Diderot, France

## Abstract

Kaiso is the first member of the POZ family of zinc finger transcription factors reported to bind DNA with dual-specificity in both a sequence- and methyl-CpG-specific manner. Here, we report that Kaiso associates with and regulates the *cyclin D1* promoter via the consensus **K**aiso **b**inding **s**ite (KBS), and also via methylated CpG-dinucleotides. The methyl-CpG sites appear critical for Kaiso binding to the *cyclin D1* promoter, while a core KBS in close proximity to the methyl-CpGs appears to stabilize Kaiso DNA binding. Kaiso’s binding to both sites was demonstrated *in vitro* using **e**lectrophoretic **m**obility ***s***hift **a**ssays (EMSA) and *in vivo* using **Ch**romatin **i**mmuno**p**recipitation (ChIP). To elucidate the functional relevance of Kaiso’s binding to the *cyclin D1* promoter, we assessed Kaiso overexpression effects on a minimal *cyclin D1* promoter*-*reporter that contains both KBS and CpG sites. Kaiso repressed this minimal *cyclin D1* promoter-reporter in a dose-dependent manner and transcriptional repression occurred in a KBS-specific and methyl-CpG-dependent manner. Collectively our data validates *cyclin D1* as a Kaiso target gene and demonstrates a mechanism for Kaiso binding and regulation of the *cyclin D1* promoter. Our data also provides a mechanistic basis for how Kaiso may regulate other target genes whose promoters possess both KBS and methyl-CpG sites.

## Introduction

In the past decade, increasing evidence has revealed an important role for epigenetic modifications such as DNA methylation in the regulation of gene expression, reviewed in [1], [2]. Specifically, the methylation of cytosine bases in CpG-dinucleotides within gene promoters plays a key role in transcriptional repression of various target genes that are implicated in many human diseases including cancer [1], [2], [3]. These promoter-associated methylated CpG-dinucleotides are recognized and bound by proteins that can distinguish between methylated and non-methylated CpG sites [4]. Until recently, the vast majority of methyl-DNA binding proteins were characterized by the presence of a **m**ethyl-DNA **b**inding **d**omain (MBD) [4]. However, several recent studies revealed that other protein families also possess methyl-DNA binding abilities, reviewed in [4], [5]. For example, the novel **Po**x virus and **z**inc **f**inger (POZ-ZF) transcription factor Kaiso and its Kaiso-like relatives, ZBTB4 and ZBTB38, recognize and bind methylated CpG-dinucleotides and repress transcription via these methylated-CpG sites [5], [6], [7]. However Kaiso, ZBTB4 and ZBTB38 all lack an MBD [6], [7]. Interestingly, Kaiso and ZBTB4 also bind DNA in a sequence-specific manner via the consensus **K**aiso **b**inding **s**ite (KBS; TCCTGCNA, where N is any nucleotide) and this distinguishes them as unique dual-specificity transcription factors [6], [8]. Of these three proteins, Kaiso is the best characterized and represses target genes that are causally linked to vertebrate development and tumorigenesis [5], [9], [10], [11], [12], [13], [14].

Kaiso was originally discovered as a binding partner for the Src kinase substrate and cell adhesion catenin cofactor p120^ctn^ [15]. This interaction was reminiscent of the β-catenin-TCF interaction that plays a crucial role in canonical WNT signaling [16], [17]; indeed, we and others found that Kaiso represses a subset of Wnt target genes while p120^ctn^’s interaction with Kaiso relieves Kaiso-mediated transcriptional repression [9], [10], [12]. Kaiso is a member of the POZ-ZF family of transcription factors that play important roles in vertebrate development and tumorigenesis [18]. Structurally, Kaiso possesses the characteristic protein-protein interaction POZ domain at its N-terminus and three C_2_H_2_-type DNA-binding zinc fingers at its C-terminus [15]. It is through these zinc fingers that Kaiso binds DNA with dual-specificity via the sequence-specific KBS or methylated CpG-dinucleotides to exert its gene regulatory effects [11], [12], [14], [19]. For example, Kaiso represses *Wnt 11* [9] and the matrix metalloprotease gene *matrilysin* in a sequence-specific manner [12], whereas it represses the *metastasis-associated gene 2* (*MTA2*) in a methyl CpG-dependent manner [14]. The importance of the methylation-dependent versus sequence-specific transcriptional regulation by Kaiso remains controversial. Thus, we initiated studies to characterize the Wnt signaling target and cell cycle regulator gene *cyclin D1* as a putative Kaiso target gene, because its promoter possessed both sequence-specific KBS’s and CpG-dinucleotide rich regions. Although previous studies in *Xenopus* and human lung tumor cells have implicated *cyclin D1* as a putative Kaiso target gene [10], [20], the direct mechanism(s) by which Kaiso binds and negatively regulates *cyclin D1* expression remain unknown.

Here we demonstrate that Kaiso binds directly to the *cyclin D1* promoter in a KBS sequence-specific or methyl-CpG-dependent manner. ChIP assays confirmed an endogenous association between Kaiso and the *cyclin D1* promoter, and our minimal promoter-reporter assays demonstrate that Kaiso represses *cyclin D1* promoter-driven luciferase activity. Importantly, Kaiso’s ability to repress the minimal *cyclin D1* promoter-reporter was abolished upon mutation of the KBS and in the absence of CpG methylation. Collectively, our data demonstrate that Kaiso transcriptionally represses the cell cycle regulator *cyclin D1*, and suggest that *cyclin D1* is a *bona fide* Kaiso target gene regulated by Kaiso’s dual-specificity mechanisms. Our study also shows that Kaiso’s sequence-specific and methylation-dependent DNA binding and transcriptional regulation may not be mutually exclusive events but instead may function to fine-tune gene expression and/or expand the repertoire of genes regulated by Kaiso.

## Materials and Methods

### Cell Culture

Human MCF7 (breast carcinoma) and HCT 116 (colon carcinoma) cells were purchased from ATCC (Manassas, VA) and grown in Dulbecco’s Modified Eagles medium (DMEM) (Hyclone) supplemented with 4 mM L-glutamine, 100 U/ml penicillin, 100 µg/ml streptomycin (Invitrogen, Life Technologies, Grand Island, NY), 10% fetal bovine serum (Hyclone) and 0.5 µg/ml fungizone (Invitrogen/Life Technologies). The cells were grown at 37°C and 5% CO_2_ in a humidified incubator. For 5-azacytidine treatment, cells were incubated in 5 µM 5-azacytidine (Sigma Aldrich) in serum supplemented DMEM for 72 hours. Due to the short half-life of 5-azacytidine in solution, fresh serum supplemented DMEM with 5-azacytidine to a final concentration of 5 µM was replenished every 24 hours during the 72-hour incubation period.

### Electrophoretic Mobility Shift Assay (EMSA)

Double-stranded oligonucleotides spanning the appropriate KBS or CpG sites in the *cyclin D1* promoter were annealed, radiolabelled and purified as previously described [21]. The -1067 KBS probe (5′ TTATGC**CG**GC*TCCTGCCA*GCCCCCTCA**CG**C 3′) contained the consensus Kaiso binding site (*TCCTGCNA*, underlined and italicized) and two CpG-dinucleotides (**bold**) while the +69 KBS probe (5′ CTGT**CG**G**CG**CAG*TAGCAG* **CG**AGCAGCAGAG 3′) contained the core KBS (*CTGCNA*) and three CpG-dinucleotides (**bold**). The *cyclin D1* promoter-derived CpG oligonucleotide sequences used in this study are listed in Table 1. In brief, the CpG and +69 KBS oligonucleotides were methylated using *Sss1* methyltransferase according to the manufacturer’s protocol (New England Biolabs - NEB, Ipswich, MA). The oligonucleotides were end-labeled at 37°C for 45 minutes with [γ-^32^P] ATP using polynucleotide kinase (NEB). Both un-methylated and methylated radiolabelled oligonucleotides were purified on a TE-10 column (Clontech, Mountain View, CA) and radioactivity was quantitated on a Tri-Carb 2900TR scintillation analyzer (Perkin Elmer). 30,000 cpm of each labeled probe was incubated with the specified bacterially-expressed GST-Kaiso fusion proteins in 1X binding buffer (25 mM HEPES, 100 mM KCl, 1 mM EDTA, 10 mM MgCl_2_, 0.1% NP40, 5% glycerol & 1 mM DTT) on ice for 30 minutes followed by incubation at room temperature for 30 minutes. Each reaction was loaded onto a 4% polyacrylamide gel in 0.5X TBE (45 mM Tris Borate, 1 mM EDTA) and electrophoresed for 2.5 hours at 200 V. The gel was dried at 80°C for 1.5 hours and exposed to XAR film at −80°C overnight.

**Table 1:** *cyclin D1*-derived oligonucleotides used in EMSA to assess Kaiso binding.

Probe Name	Oligonucleotide Sequence	# CpGs
−1067 KBS	TTATGC**CG**GC***TCCTGCCA***GCCCCCTCA**CG**C	2
+69 KBS	CTGT**CG**G**CG**CAG***TAGCAG*** **CG**AGCAGCAGAG	3
CpG1	G**CG**GGGGAGGGGG**CGCG**GGAGGAATTCACC	2
CpG2	**CG** TTCTTGGAAATG**CG**CCCATTCTGC**CG**GC	3
CpG3	TATGGGGTGT**CG**C**CGCG**CCCCAGTCACCCC	2
CpG4	GC**CG**CAGGGCAGG**CGCG**G**CG**CCTCAGGGAT	3
CpG5	CC**CG**G**CG**TTTGG**CG**CC**CGCG**CCCCCTCCCC	4
CpG6	GCCCCCTCCCCCTG**CG**CC**CG**CCCC**CG**CCCC	3
CpG7	CAGAGGGCTGT**CG**G**CG**CAG***TAGCAG*** **CG**AGC	3
CpG8	GAGGGGCAGAAGAG**CGCG**AGGGAG**CGCG**GG	2

Ten oligonucleotides were synthesized from different regions of the *cyclin D1* promoter and used in EMSA experiments to elucidate Kaiso binding. The CpGs are bolded while the KBSs are bolded and italicized (i.e. −1067KBS, +69KBS and CpG7).

### *cyclin D1* Promoter Sub-cloning and *in vitro* Methylation

The minimal *cyclin D1* promoter region (−1748 to +164) was PCR amplified and sub-cloned upstream of the *Gaussia* luciferase gene in the pGLuc Basic vector (NEB) using *Kpn1* and *BamH1* sites. This *cyclin D1* promoter-reporter luciferase construct was designated as the −1748*CD1* wild type reporter, and contained the −1067 and +69 core KBSs in addition to multiple CpG sites. The KBS sequences located at positions −1067 (designated 1) and +69 (designated 2) were mutated via site-directed mutagenesis. The mutations were confirmed by sequencing (Mobix Facility, McMaster University) and the resulting plasmid called −1748*CD1* KBS (1, 2) mutant. The reporter plasmids were purified from dam^−/^dcm^−^ bacteria and then methylated by treating with the methyl donor S-adenosylmethionine (NEB) in the presence of bacterial *Sss1* CpG methyltransferase (NEB). Briefly, 50 µg of each plasmid DNA was incubated with 200U of *Sss1* methyltransferase in a 250 µL reaction that contained 640 µM S-adenosyl methionine and 1X NEB buffer. The reactions were incubated at 37°C for 2 hours, after which the enzyme was inactivated at 65°C for 20 minutes. The methylated DNA samples were purified by standard phenol-chloroform extraction and ethanol precipitation. CpG-methylated plasmids were digested with the methylation-resistant restriction enzyme *HpaII* to confirm complete methylation. The pGluc-Basic vector was used as a negative control while the pGluc-1748*CD1* wild type and mutated reporters were used to assess Kaiso’s regulation of the *cyclin D1* promoter via the KBS and methylated CpG sites.

### Transient Transfection and Luciferase Assays

MCF7 cells were seeded at 2.5×10^5^ cells/mL into 6-well dishes and incubated for at least 12 hrs until the cells were approximately 50–60% confluent. Each well was transfected with 600 ng of reporter DNA plasmid (pGLuc-Basic, pGLuc-Basic wild type −1748*CD1* or pGLuc-Basic −1748*CD1* KBS (1,2) mutant), 500 ng of pRSV/β-galactosidase internal control and various amounts of effector plasmids (pcDNA3 empty, pcDNA3 human Kaiso, or pRS-Kaiso) by diluting the DNA in 150 mM NaCl and mixing gently before adding 10 equivalents (∼17 µl) of ExGen-500 reagent (Fermentas, Burlington, ON). The mixture was gently vortexed, and incubated without disturbing at RT for 15 minutes to allow transfection complex formation. The complexes were then added drop-wise to the cells in fresh serum-supplemented DMEM medium before incubating the cells for 3 hours at 37°C with 5% CO_2_, after which the reagent was aspirated and replaced with 2 mL of fresh DMEM. 24 hours post-transfection, 25 µL of the culture medium was assayed for luciferase activity with 50 µL of *Gaussia* luciferase substrate (NEB) on an LB luminometer (Thermo Fisher). Luciferase activity was recorded as relative light units (RLU’s) and normalized for transfection efficiency using the internal control β-galactosidase activity for each experimental and control sample condition.

### Chromatin Immunoprecipitation (ChIP)

MCF7 and HCT 116 cells were grown to ∼80% confluency and cross-linked with 1% formaldehyde in DMEM medium. The cells were placed on a belly dancer and gently shaken for 10 minutes at room temperature. Formaldehyde fixation was stopped by adding 1 M glycine to a final concentration of 125 mM and the cells rocked for 5 minutes at room temperature. The cells were washed twice with 5 mL of ice-cold 1X PBS, and harvested by scraping in 1 mL PBS containing complete Mini Protease Inhibitor Cocktail Tablet (Roche, Mannheim, Germany). The cells were collected in 15 mL conical tubes and pelleted by centrifugation at 4°C for 5 minutes at 2,000 rpm and the supernatants aspirated. Cell pellets were re-suspended in 2 mL of ice-cold ChIP lysis buffer (5 mM PIPES pH 8.0, 85 mM KCL, 0.5% NP-40, with protease inhibitors) and dounced ten times with a homogenizer, before incubating on ice for 15 minutes. The lysates were centrifuged at 5,000 rpm for 5 minutes at 4°C and the nuclear pellet resuspended in 250 µL of Nuclear lysis buffer (50 mM Tris-Cl pH 8.1, 10 mM EDTA, 1% SDS, with protease inhibitors). After incubating on ice for 10 minutes, the nuclear pellets were sonicated at 90% duty, 5% power for 5 rounds of 15-second pulses to achieve sheared chromatin fragment lengths of ∼100–1000 base pairs. The lysates were cleared by centrifugation at 14,000 rpm for 10 minutes at 4°C and the supernatant transferred to new microfuge tubes. 7.5 µg of chromatin was pre-cleared by incubating end-over-end for 1 hour at 4°C with 5 µL of rabbit IgG (Abcam, Cambridge, MA) in a 500 µL reaction. Fifty µL of salmon sperm-blocked Protein A beads was added to the pre-cleared lysate and rotated as above before centrifuging at 5,000 rpm for 3 minutes. The supernatant was subjected to immunoprecipitation with 4 µg Kaiso 6F monoclonal antibody [22], 2 µg Histone H3 polyclonal antibody (Abcam) or negative control mouse IgG antibody (Active Motif, Carlsbad, CA) at 4°C and rotated end-over-end overnight. The immunoprecipitated samples were centrifuged at 13,000 rpm for 2 minutes at 4°C before 50 µL of Protein-A rabbit-anti-mouse bridge or Protein-A beads (depending on antibody isotype used for IP) was added to each immunoprecipitated supernatant sample. Samples were rotated end-over-end at 4°C for 1 hour and the precipitated samples washed six times (1X with 1 mL of RIPA buffer for 10 minutes at 4°C, 1X with high salt buffer for 10 mins, 1X with LiCl buffer for 5 minutes and 2X with TE buffer for 10 minutes each). After removing the supernatant, 300 µL of 1X TE buffer and 1.5 µL of RNase A (10 mg/mL) was added to the immunoprecipitate and 10% input samples before incubating for 30 minutes at 37°C. 15 µL of 10% SDS and 3.75 µL of proteinase K (20 mg/mL) were added and the samples incubated at 37°C for a minimum of 4 hours. The samples were then reversed cross-linked overnight at 65°C and DNA purified using standard phenol-chloroform extraction and ethanol precipitation. The DNA was resuspended in 50 µL of sterile dH_2_O and used for PCR amplification.

### PCR Amplification

Two microliters of recovered DNA from each chromatin immunoprecipitated sample was used in a PCR reaction that contained 1X PCR buffer, 1.5 mM MgCl_2_, 0.3 mM dNTPs, 0.4 mM forward and reverse primers (-1067 KBS-Forward: 5′-TTTACATCTGCTTAAGTTTGCG-3′ & -1067 KBS-Reverse 5′-TTAGAATTTGCCCTGGGACT-3′, +69 KBS-Forward: 5′-CACACGGACTACAGGGGAGTT-3′ & +69 KBS-Reverse: 5′-CTCGGCTCTCGCTTCTGCTG-3′, CpG5-Forward: 5′-TTTGCATTTCTATGAAAACCGG-3′, & CpG5-Reverse 5′-GCAACTTCAACAAAACTCCC-3′, and CpG8-Forward: 5′-ACACGGACTACAGGGGAGTTTTG-3′ & CpG8-Reverse: 5′-ATTTCGAACCCCTGGGGAGG-3′ and negative control-Forward: 5′-CCCTCGGTGTCCTACTTCAA-3′ & negative control-Reverse 5′-CACCACGGCAAACTTCAAAG-3′), 0.5 µl of Taq polymerase (Invitrogen/Life Technologies), and sterile water to a final volume of 25 µl. The PCR conditions were as follows: initial denaturation at 95°C for 5 minutes, followed by 36 cycles of denaturation at 95°C for 1 minute, annealing at 50°C for 1 minute (for the -1067 KBS), 53°C for 1 minute (for the +69 core KBS), 53.6°C for 45 seconds (for CpG5) or 58°C for 45 seconds (for CpG8) with extension at 72°C for 30 seconds, and a final extension at 72°C for 45 seconds. 10 µL of each PCR reaction were loaded onto a 1.2% agarose gel with 0.5 µg/ml EtBr, electrophoresed at 120 V for 25 minutes in 1X TAE solution and the gel imaged.

### Western Blot

HCT116 and MCF7 cells were washed twice with 5 mL of cold 1XPBS and lysed with 500 µL lysis buffer containing 0.5% NP-40, 0.5% Na_3_VO_4_ and complete mini protease inhibitor cocktail tablet (Roche). Lysates were centrifuged at 13,000 RPM for 15 minutes at 4°C and the pellet discarded. 10 µg of total protein was denatured in 2X **L**aemmli **s**ample **b**uffer (LSB) by boiling for 5 minutes. Equal amounts of protein were separated by SDS-**p**oly**a**crylamide **g**el **e**lectrophoresis (PAGE) and transferred onto nitrocellulose membrane. Membranes were blocked for 1 hour at room temperature with 3% skim milk in 1X **T**ris **B**uffered **S**aline (TBS; pH 7.4). The membranes were then incubated overnight at 4°C with primary antibodies at the following dilutions: anti-Kaiso rabbit polyclonal antibody (1∶30,000); anti-Cyclin D1 rabbit monoclonal antibody (Cell Signaling; 1∶1000); anti-β-actin mouse monoclonal antibody (Sigma Aldrich; 1∶5000). Membranes were washed once for 30 minutes and then 4 times for 5 minutes with 1XTBS, pH 7.4, followed by incubation with either donkey anti-mouse or goat anti-rabbit **h**orse**r**adish **p**eroxidase (HRP)-conjugated secondary antibodies (1∶40 000) for 2 hours at room temperature with rocking. Membranes were washed as described, and then processed and visualized using the Enhanced Chemiluminescent System (Amersham Biosciences) according to the manufacturer’s protocol.

### MTT Cell Proliferation Assay

Cells were seeded in 96-well plates in triplicate in 100 µL of serum-supplemented media. 24 hours after seeding, 20 µL thiazolyl blue tetrazolium bromide (Sigma Aldrich) in dH_2_O was added to the media in each well to a final concentration of 0.5 mg/mL. Cells were incubated for 4 hours in a 5% CO_2_, humidified incubator. Following incubation, media was aspirated from wells (without disturbing purple crystals at bottom) and 100 µL per well DMSO was added to cells to solubilize formazan crystal product. Crystals were allowed to dissolve for 5–10 minutes and absorbance read at 570 nm using a spectrophotometer. Growth of HCT 116 pRS-empty and HCT 116 pRS-Kaiso was plotted relative to the HCT 116 parental cell line.

## Results

### Kaiso Binds the *cyclin D1* -1067 Promoter Region in a KBS-specific Manner

**Figure 1:**
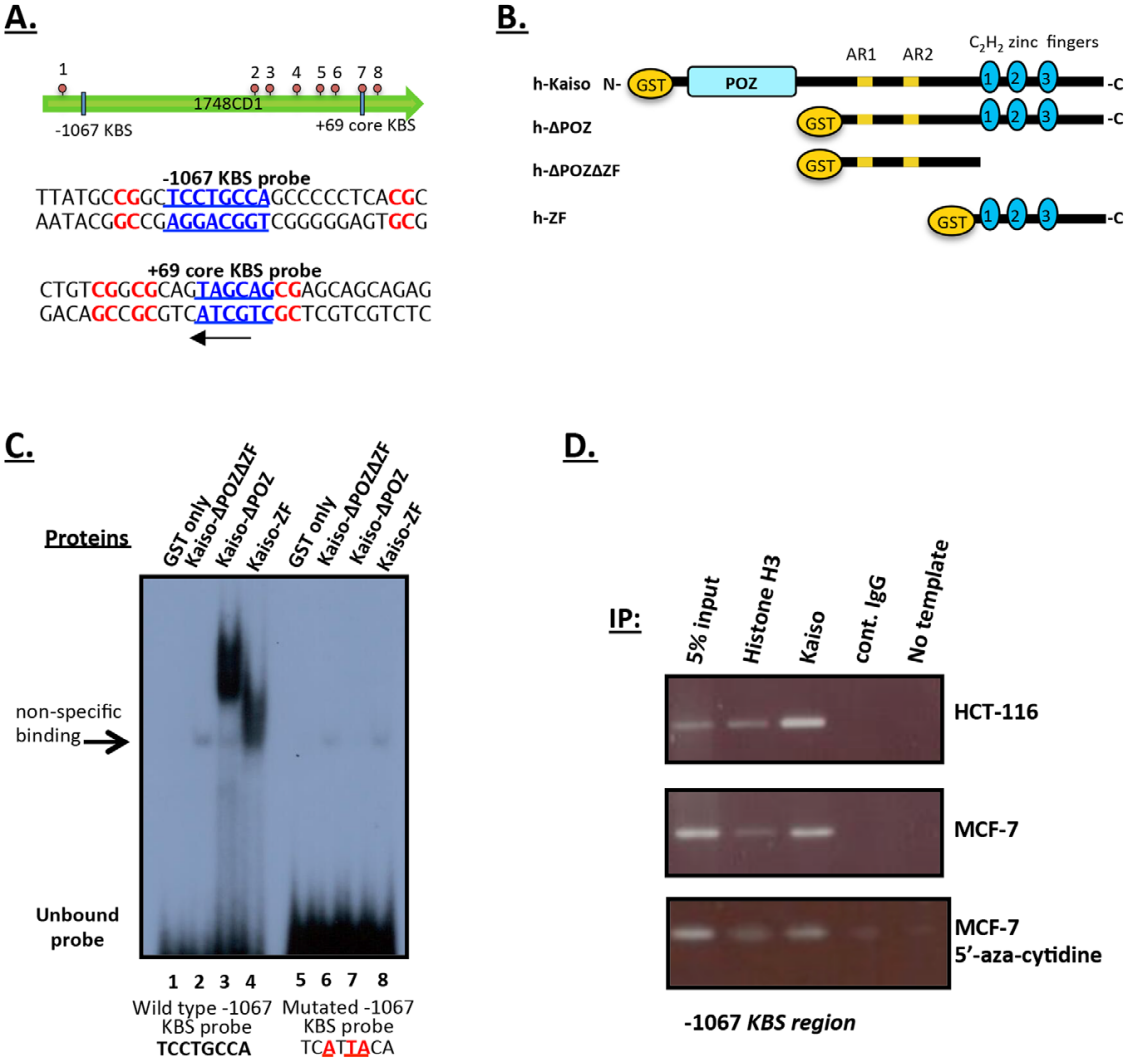
Kaiso binds specifically to the −1067 KBS site of the *cyclin D1* promoter *in vitro* and *in vivo*. (**A**) Schematic representation of the ∼1748 *cyclin D1* promoter fragment showing the −1067 KBS, the +69 core KBS (in blue and underlined) and some of the CpG sites in red. Red circles represent CpG sites analyzed in this study. (**B**) Schematic of GST-Kaiso fusion proteins used in this study. The various GST-Kaiso fusion proteins were expressed in bacteria before purification using GST beads. The N-terminal GST-moiety, the Kaiso-POZ domain and three zinc fingers are indicated. (**C**) GST-Kaiso proteins bound the wild type radiolabelled −1067 oligonucleotide probe in a KBS-specific manner. The negative controls, GST alone and GST-KaisoΔPOZΔZF, lacking the POZ and ZF domain did not bind the probe. None of the GST-Kaiso fusion proteins bound the −1067 probe when the KBS was mutated. (**D**) ChIP analysis of the *cyclin D1* promoter in HCT 116 and MCF7 cells revealed that Kaiso specifically associates with the *cyclin D1* promoter −1067 KBS region.

**Figure 2:**
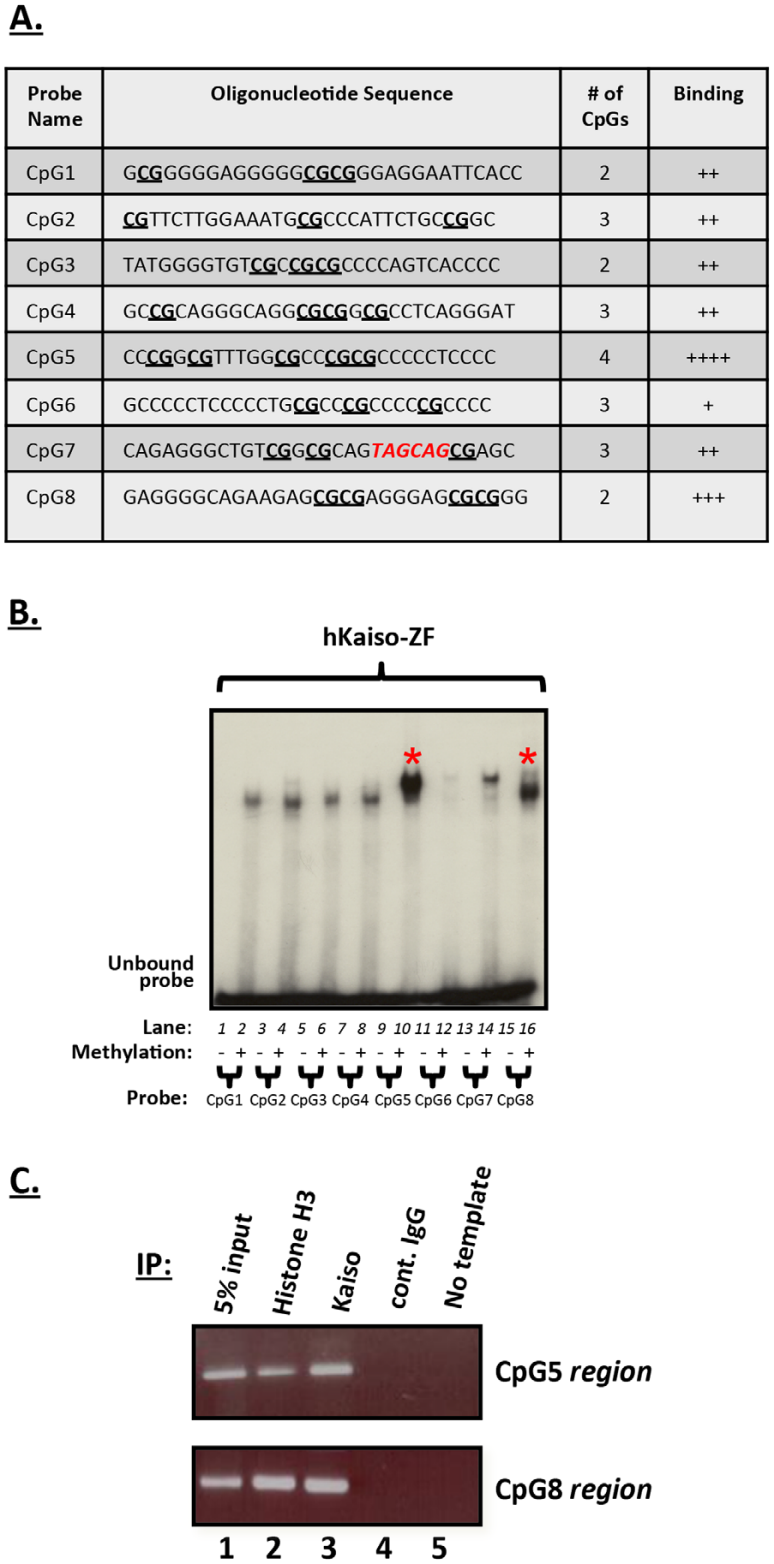
Kaiso binds specifically to methyl-CpG-dinucleotides in the *cyclin D1* promoter. (**A**) Summary of Kaiso binding to methyl-CpG sites in *cyclin D1* promoter-derived oligonucleotides. Eight CpG probes were synthesized from different regions of the *cyclin D1* promoter and used in EMSA experiments to elucidate Kaiso binding. The CpGs are bolded and underlined while the KBS is bolded and red. (**B**) EMSA revealed that Kaiso bound both single and consecutive CpG dinucleotides within *cyclin D1* promoter-derived oligonucleotides in a methylation-specific manner. Asterisks (*) denote very strong binding. (**C**) ChIP analysis of HCT 116 chromatin revealed that Kaiso specifically associated with the CpG5 and CpG8 sites in the *cyclin D1* promoter.

*Cyclin D1* was first postulated to be a potential Kaiso target gene after elevated *cyclin D1* mRNA levels were detected in *Xenopus laevis* embryos following *xKaiso* depletion [10]. More recently studies in lung cancer cell lines have also implicated *cyclin D1* as a Kaiso target gene [20]. However, *cyclin D1* was never validated as a *bona fide* Kaiso target gene and it was unknown whether the changes in *cyclin D1* mRNA and protein levels were a direct or indirect effect of transcriptional regulation by Kaiso. Our lab has identified numerous CpG dinucleotide-rich regions and three KBSs (at positions −2336, −1067 and +69 in the *cyclin D1* promoter, ID#:6842 from the Transcriptional Regulatory Element Database) relative to the transcriptional start site (Figure 1A). As a first step towards validating *cyclin D1* as a Kaiso target gene and determining the mechanism by which it is regulated, we examined Kaiso’s ability to bind the human *cyclin D1* promoter *in vitro*. We performed EMSA studies using various bacterially-expressed and purified GST-Kaiso fusion proteins and nine oligonucleotides that individually corresponded to the KBS found at position -1067 and various CpG rich regions of the *cyclin D1* promoter (Figures 1, 2 & S1). The −1067 KBS oligonucleotide used in Figure 1 possessed the full KBS (TCCTGCNA) and two CpG sites while one of the CpG oligonucleotides (CpG7) used in Figure 2 contained a core KBS (CTGCNA) and three CpG sites (see Figure 2).

We employed GST-Kaiso fusion proteins lacking the Kaiso POZ domain for our EMSA studies, since we and others have found that the presence of the POZ domain in most full-length POZ-ZF proteins resulted in weak or no association with DNA *in* *vitro,* [23] and our unpublished data. In repeated experiments, we found that GST-Kaiso-ΔPOZ (lacking the N-terminal POZ domain) and GST-Kaiso-ZF domain could bind the −1067 KBS region of the *cyclin D1* promoter (Figure 1C, lanes 3 & 4 ). As expected, no binding was observed with the GST alone or GST-Kaiso-ΔPOZΔZF negative controls (Figure 1C, lanes 1 & 2). To confirm that Kaiso was binding the −1067 region in a KBS-specific manner, three point mutations were introduced into the core KBS (CTGCNA to **A**T**TA**CA) sequence. When this mutated oligonucleotide (−1067 mut.) was tested in EMSA, Kaiso DNA binding was completely abolished (Figure 1C, lanes 7 & 8). This confirmed that Kaiso was binding directly to the −1067 *cyclin D1* promoter region in a KBS-specific manner.

To confirm that Kaiso bound the −1067 KBS region of the *cyclin D1* promoter endogenously, we next performed ChIP experiments using chromatin isolated from MCF7 and HCT 116 cells, which express moderate to high levels of Kaiso respectively, and immunoprecipitated the DNA-protein complexes with the Kaiso-specific monoclonal antibody 6F [22]. PCR was performed with primers that flanked the −1067 KBS region in the *cyclin D1* promoter and we repeatedly amplified fragments of ∼170 bp from MCF7 and HCT 116 chromatin samples (Figure 1D). This fragment was also present in the 5% input and Histone-H3 positive control lanes but absent in the IgG negative control and no template lanes. Interestingly, treatment of MCF7 cells with 5′-azacytidine for 3 days did not affect Kaiso’s ability to associate with the −1067 KBS region (Figure 1D). Our findings confirm that Kaiso associates with the *cyclin D1* promoter endogenously via the −1067 KBS region and suggest that this interaction may be methylation independent.

### Kaiso Binds *cyclin D1* Promoter Regions Possessing Multiple Methyl-CpG Sites

Since Kaiso is a dual-specificity DNA-binding transcription factor that also binds methylated CpG-dinucleotides [7], [19], [21] and the *cyclin D1* promoter possesses many CpG sites, we performed studies to determine whether Kaiso could bind and regulate *cyclin D1* expression via some of these CpG sites. Thus, we synthesized eight oligonucleotides corresponding to eight different CpG regions of the *cyclin D1* promoter (spanning −1504 to +102 relative to the transcriptional start site, Figure 1A). Each oligonucleotide possessed CpG-dinucleotides but some contained three single CpG-dinucleotides (*e.g.* CpG-2, -6, -7) while others possessed a combination of single and consecutive CpG-dinucleotides, (*e.g.* CpG-1, -3, -4, -5, -8) (Figure 2A). All oligonucleotide probes were methylated *in vitro* with *Sss1* methyltransferase and then individually tested for Kaiso’s ability to bind them. Using GST-Kaiso-ZF fusion proteins, we found that Kaiso bound all eight oligonucleotides with varying efficiency in a methylation-specific manner (Figure 2B). Interestingly, Kaiso bound most efficiently to probes containing two consecutive CpG and three single CpG-dinucleotides (CpG5) or two sets of consecutive CpG-dinucleotides (CpG8) (Figure 2B, lanes 10 and 16). Surprisingly, Kaiso did not bind the non-methylated CpG7 probe that possessed a core KBS and this suggested that in the context of the *cyclin D1*+69KBS region, Kaiso has a higher affinity for methyl-CpG-dinucleotides than for the KBS.

As before, we confirmed the Kaiso–methyl-CpG interaction *in vivo* using ChIP experiments with chromatin isolated from HCT 116 cells, which express high levels of Kaiso, and the Kaiso-specific monoclonal antibody 6F (Figure 2C). PCR was performed with primers that flanked the two CpG sites that showed the highest levels of Kaiso binding in EMSA (CpG5 and CpG8). We repeatedly amplified fragments of ∼233 bp and ∼197 bp corresponding to the *cyclin D1* CpG5 and CpG8 regions respectively (Figure 2C). These fragments were absent in the IgG negative control and no template lanes. Hence, our data indicate that Kaiso also associates specifically with the *cyclin D1* promoter endogenously via the CpG5 and CpG8 regions.

### Kaiso Binds Specifically to the +69 core KBS Region in a Methyl-CpG Dependent Manner

**Figure 3:**
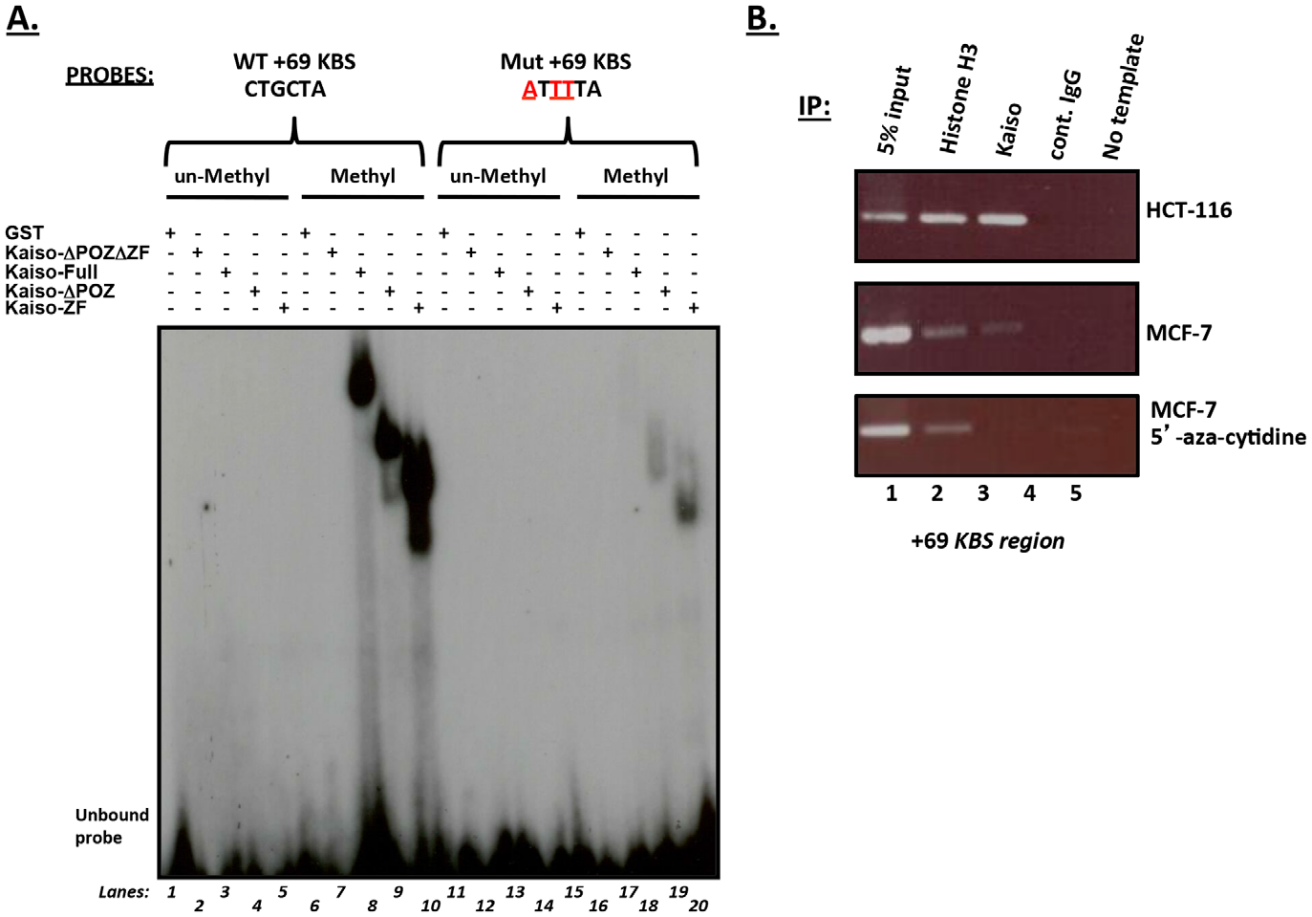
Kaiso binds the +69 core KBS region of the *cyclin D1* promoter *in vitro* and *in vivo.* (**A**) EMSA revealed that Kaiso bound the methylated *cyclin D1*+69 KBS promoter region but not the unmethylated +69 KBS probe. Kaiso also bound weakly to the methylated (but KBS mutated) +69 KBS probe compared to the wild type probe. (**B**) ChIP of the *cyclin D1* promoter in HCT 116 and MCF7 cells revealed that Kaiso specifically associated with the *cyclin D1* promoter +69 KBS region. 5′-azacytidine treatment of MCF7 cells abolished Kaiso’s association with the *cyclin D1* promoter and suggests methyl-CpG-dependent binding of Kaiso to the promoter.

Since Kaiso bound to the methylated CpG7 but not to the non-methylated CpG7 which possessed a core KBS (CTGCNA) and three single CpG dinucleotides (Figure 2B, compare lanes 13 & 14), we sought to determine the relevance of this core KBS in the *cyclin D1* promoter and whether it contributed to Kaiso’s binding to this probe. EMSA experiments were performed with an oligonucleotide named “+69 core KBS” that encompassed most of the CpG7 probe and seven additional nucleotides at the 3′ end. We included the full-length GST-Kaiso fusion protein in these EMSA experiments after determining that full-length Kaiso can bind the *cyclin D1* promoter-derived oligonucleotides, albeit weaker than the GST-Kaiso deletion mutants lacking the POZ domain. Consistent with our earlier findings, all the GST-Kaiso fusion proteins possessing the zinc finger domain bound the +69 core KBS oligonucleotide in a methylation-dependent manner but none bound the un-methylated oligonucleotide despite the presence of the core KBS sequence (Figure 3A, compare lanes 8–10 to lanes 3–5). Indeed, when the +69 core KBS “CTGCNA” was mutated to “**A**T**TT**NA” the GST-Kaiso fusion proteins still bound the methylated mutated probe (Figure 3A, lanes 19 & 20) albeit with a lower affinity than the wild type probe. This suggested that methylation is necessary and sufficient for Kaiso binding to the +69 region. However, although the core KBS does not appear to the essential for Kaiso binding to the +69 KBS region, the presence of the core KBS seems to stabilize or increase the affinity for Kaiso binding to this site (compare Figure 3A lanes 19 & 20 to lanes 9 & 10). ChIP experiments using the Kaiso-specific monoclonal antibody 6F confirmed that Kaiso associated endogenously with the *cyclin D1*+69 KBS promoter region in MCF7 and HCT 116 cells (Figure 3B). More importantly, treatment of MCF7 cells with 5′-azacytidine abolished Kaiso’s endogenous association with the +69 KBS region as demonstrated using ChIP (Figure 3B). The specificity of Kaiso binding to the −1067, +69 KBS and CpG sites of the *cyclinD1* promoter in MCF7 cells was also confirmed using primers designed to amplify a region upstream of the KBS and CpG sites (Figure S2).

**Figure 4:**
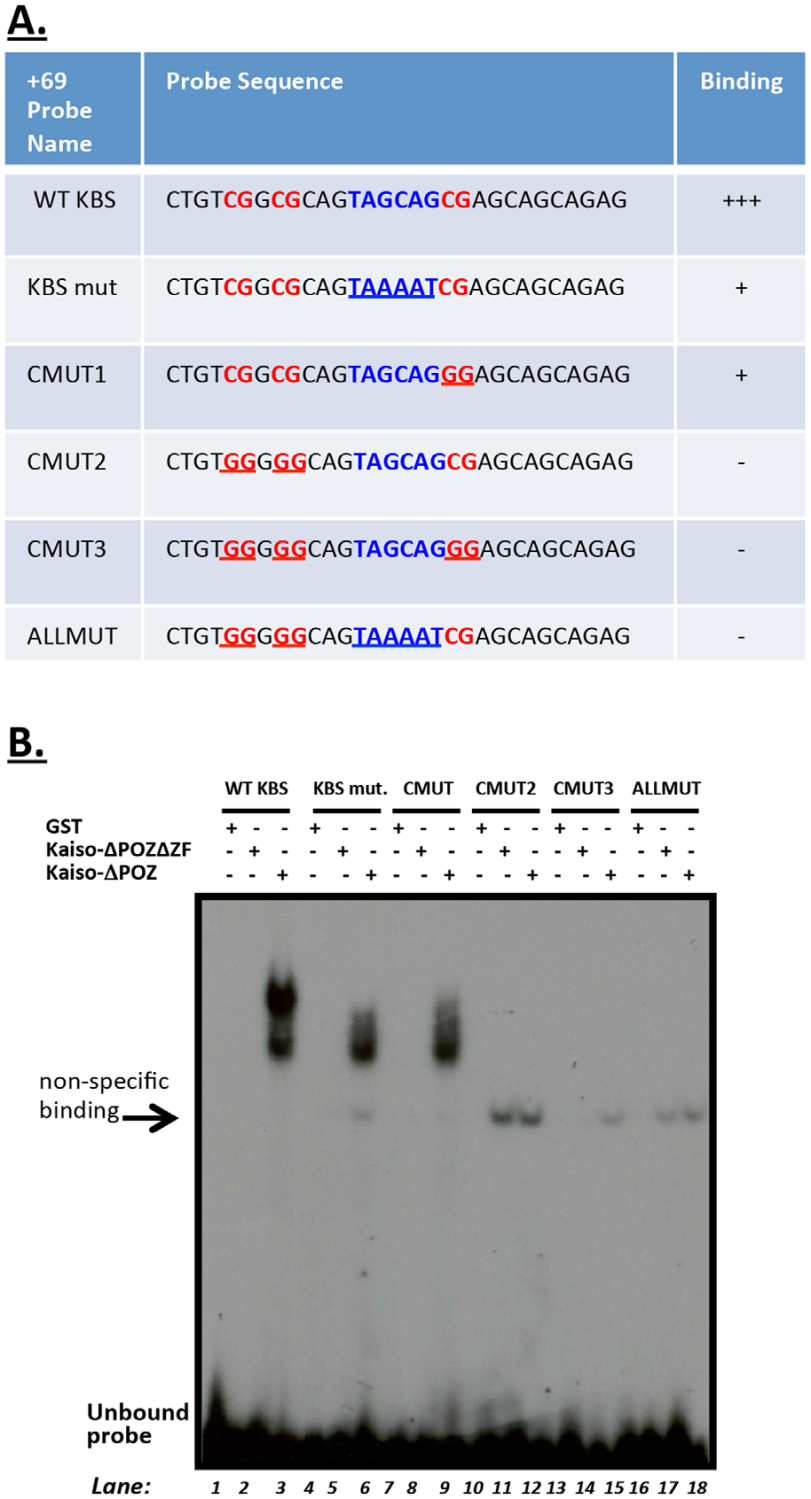
Kaiso binds the +69 core KBS region of the *cyclin D1* promoter in a methyl-CpG-specific manner. (**A**) Summary of Kaiso binding to wild type and mutated +69 core KBS *cyclin D1*-derived oligonucleotides. The CpGs (red) and KBS (blue) sites are highlighted and the mutations are underlined. (**B**) EMSA showed that Kaiso binding to the *cyclin D1*+69 KBS promoter region requires at least two intact methyl-CpG dinucleotides but not an intact KBS site.

Since some Kaiso binding was retained with the +69 KBS mutant methylated probe, we created four additional mutated probes to determine which CpG dinucleotide sites were essential for the Kaiso-DNA interaction (Figure 4A). The +69 CMUT1 (mutated one 3′ CpG to GG but with intact KBS), +69 CMUT2 (mutated the two 5′ CpGs to GGs, with intact KBS and 3′ CG), +69 CMUT3 (mutated all three CpG sites to GGs but with intact KBS) and +69 ALLMUT (mutated all three CpGs and the KBS) methylated probes were incubated with GST-Kaiso-ΔPOZ fusion proteins. GST-Kaiso-ΔPOZ bound the methylated +69 KBS-mut probe similarly to that of the +69 CMUT1 probe, but with lower affinity than the wild type probe (Figure 4B, compare lanes 6 & 9 to 3). Since Kaiso did not bind the +69 CMUT2, +69 CMUT3 or +69 ALLMUT probes (Figure 4B, lanes 10–18), this suggests that the two CpG sites immediately upstream of the KBS are necessary for Kaiso binding to the *cyclin D1*-promoter-derived oligonucleotides and supports our 5′-azacytidine ChIP experiment (Figure 3B). Taken together, our data suggest that Kaiso’s binding to the +69 KBS region is methyl-CpG-dependent and not KBS-specific. We further confirmed the specificity of Kaiso binding to the methylated +69 core KBS probe via cold competition assays with excess unlabelled probes (data not shown).

### Kaiso Represses Transcription from the *cyclin D1* Minimal Promoter in a KBS-specific Manner

**Figure 5:**
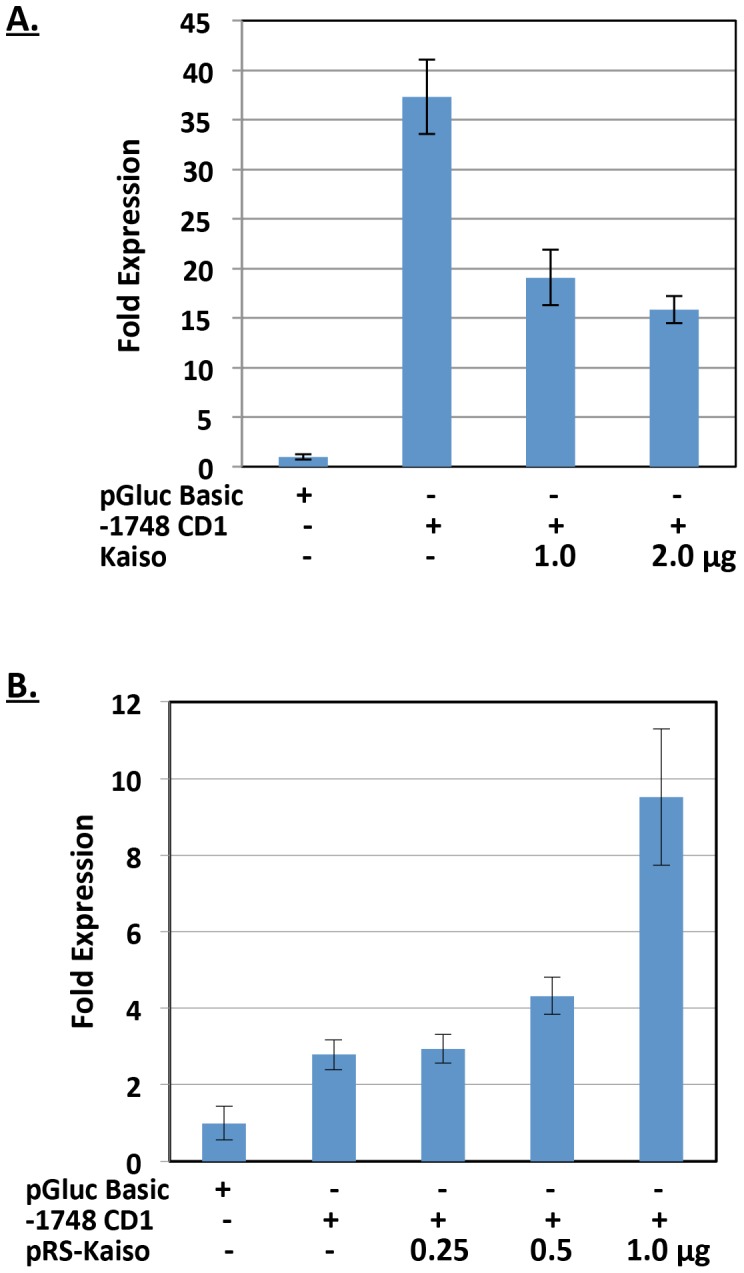
Kaiso represses expression of a minimal *cyclin D1* promoter-reporter. (**A**) Kaiso overexpression decreased luciferase expression from the minimal *cyclin D1* promoter-reporter in a dose-dependent manner in MCF7 cells. (**B**) Depletion of endogenous Kaiso caused de-repression of the minimal *cyclin D1* promoter-reporter in MCF7 cells.

After determining that Kaiso bound the *cyclin D1* promoter region with dual-specificity (*i.e.* via the sequence-specific KBS and via methyl-CpG sites), we next assessed Kaiso’s ability to regulate luciferase expression under control of a minimal *cyclin D1* promoter. Transfection of MCF7 cells with the unmethylated *cyclin D1* promoter-reporter (−1748 CD1), containing two KBSs and multiple CpG sites, resulted in an ∼35-fold increase in luciferase reporter activity compared to the pGluc-Basic negative control vector lacking the *cyclin D1* promoter region (Figure 5A). Co-transfection of the −1748 CD1 promoter-reporter and a Kaiso expression plasmid abrogated this response and resulted in a dose-dependent decrease in luciferase activity (Figure 5A). A similar trend was observed in HCT 116 cells (data not shown). To confirm that transcriptional repression was attributed to Kaiso, we depleted endogenous Kaiso with Kaiso-specific siRNA. Increasing amounts of Kaiso-specific siRNA resulted in dose-dependent de-repression of the reporter gene (Figure 5B), and confirmed that Kaiso was negatively regulating the minimal *cyclin D1* promoter. Importantly, since the *cyclin D1* promoter-reporter plasmid was propagated in dam^−/^dcm^−^ bacteria, the CpG sites were un-methylated. Thus it appears that Kaiso-mediated transcriptional repression of the *cyclinD1* promoter-reporter was occurring via the sequence-specific KBS sites and not the CpG sites.

### Kaiso Represses Transcription from the Minimal *cyclin D1* Promoter in a Methyl-CpG-specific Manner

**Figure 6:**
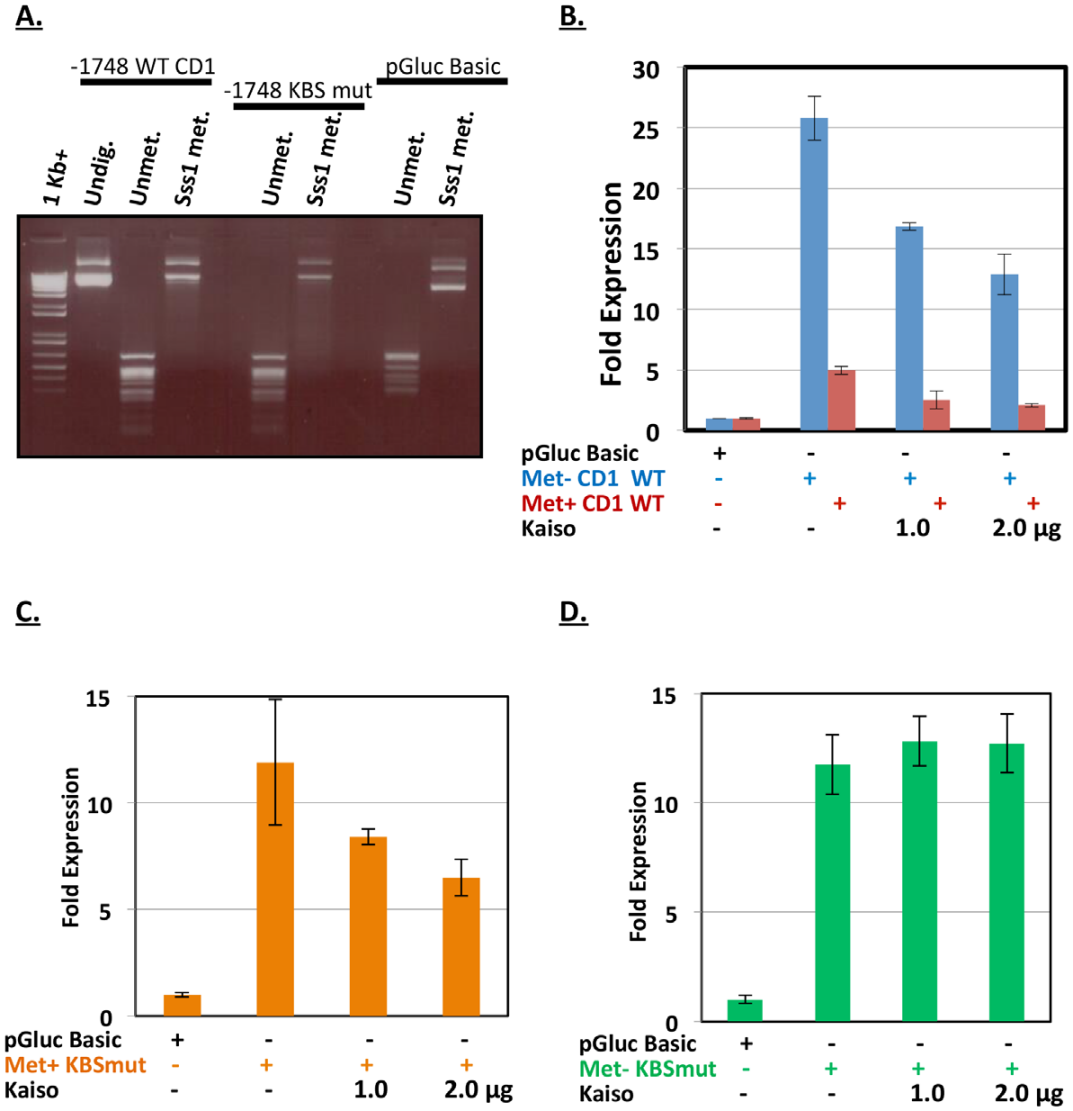
Kaiso represses expression of the minimal *cyclin D1* promoter-reporter in a KBS and methyl-CpG dependent manner. (**A**) Reporter plasmid methylation was confirmed by digesting the plasmid DNA with the CpG-methylation specific restriction enzyme *HpaII*. (**B**) Kaiso overexpression caused a dose-dependent decrease in luciferase gene expression from the minimal *cyclin D1* promoter reporter possessing active KBS but devoid of methyl-CpG sites (blue bars). Similarly, a dose-dependent decrease was observed when the KBS and CpG sites were both active (red bars). (**C**) Kaiso overexpression caused dose-dependent repression of luciferase activity in the presence of active methyl-CpG sites and mutated KBSs. (**D**) Ectopic Kaiso expression had little to no effect on the reporter construct when both the KBS and CpG sites were inactivated.

We next examined how a change in the methylation status of the promoter may affect Kaiso’s ability to regulate expression of the minimal *cyclin D1* promoter-reporter. Thus, the −1748 CD1 promoter-reporter construct was *in vitro* methylated using *Sss1* methyltransferase prior to transfection. CpG methylation of the plasmid was confirmed by restriction digest with the methylation-resistant enzyme *HpaII* (Figure 6A *)*. Transfection of the unmethylated −1748*CD1* wild-type promoter-reporter construct resulted in more than 25-fold increase in luciferase activity compared to the control pGluc-Basic vector, while its methylated counterpart only exhibited an ∼3.5-fold increase (Figure 6B). This is consistent with the notion that methylation of promoter regions is involved in gene silencing. However, when the methylated or unmethylated −1748 CD1 promoter-reporters were individually co-transfected with Kaiso, a similar two-fold decrease in luciferase activity was observed for both promoters tested (Figure 6B, compare 2.0 µg Kaiso for each reporter). This data suggests that Kaiso’s ability to repress the *cyclin D1* promoter is via at least three distinct mechanisms: (1) via binding to the KBS, (2) via binding to methylated CpG sites, or (3) via combinatorial use of both KBS and CpG sites. To further delineate Kaiso’s mechanism(s) of transcriptional repression of the *cyclin D1* promoter-reporter, we mutated the KBSs and assessed luciferase activity from the unmethylated and methylated mutant reporters. The methylated but KBS mutated promoter-reporter (Met+KBSmut) exhibited a dose-dependent decrease in luciferase activity upon ectopic Kaiso expression (Figure 6C) while its unmethylated and KBS mutated counterpart (Met-KBSmut) remained relatively unchanged (Figure 6D). Together our data suggests that Kaiso regulates *cyclin D1* via its dual-specificity DNA-binding.

### Kaiso Depletion Increases HCT116 Cell Proliferation and cyclinD1 Protein Levels

**Figure 7:**
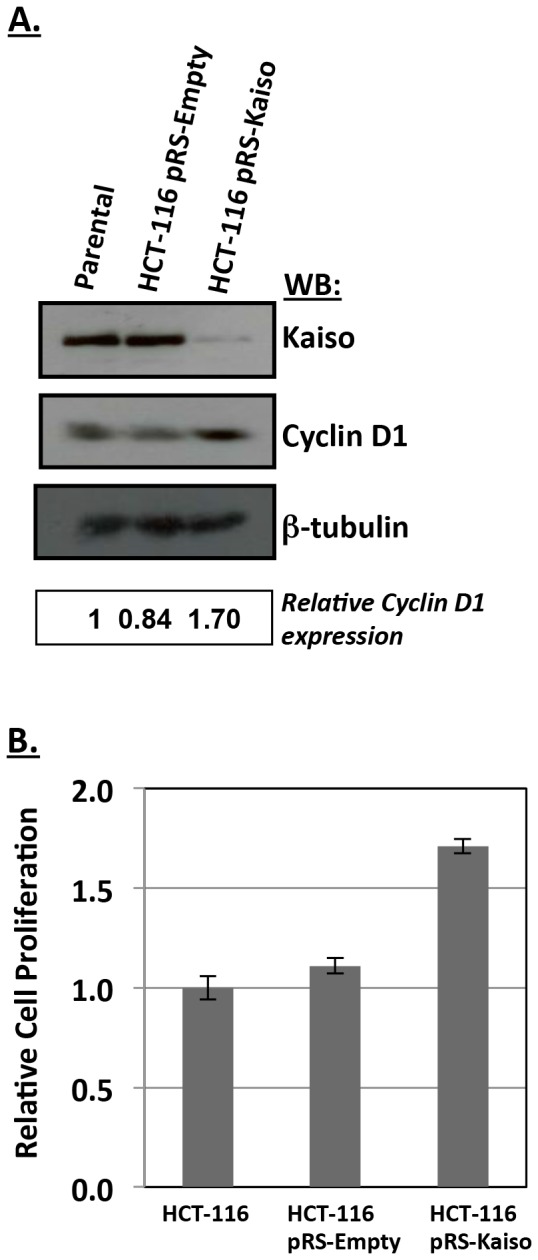
Kaiso depletion alters cyclin D1 expression and cell proliferation in HCT116 cells. (**A**) Depletion of endogenous Kaiso with Kaiso-specific siRNA resulted in an ∼ 1.7-fold increase in cyclin D1 protein levels in HCT116 cells. (**B**) Kaiso depletion in HCT116 cells resulted in an ∼ 2-fold increase in cell proliferation.

As a first step toward examining Kaiso’s potential role in cell cycle regulation we examined cyclin D1 protein levels by western blot analysis of Kaiso-depleted HCT 116 colon carcinoma cell lysates. Similar to Jiang *et al*. (2012), we found that Kaiso depletion resulted in increased cyclin D1 protein levels (Figure 7A). Conversely, transient overexpression of Kaiso in MCF7 cells resulted in decreased cyclinD1 protein levels (Figure S3). More importantly, the Kaiso-depleted cells displayed an ∼2-fold increase in cell proliferation compared to the parental and control vector HCT 116 cells in three independent trials (Figure 7B). The increased cell proliferation observed in the HCT 116 Kaiso-depleted cells strengthens our hypothesis that *cyclin D1* is a *bona fide* Kaiso target gene.

## Discussion

### Kaiso Binds and Represses the *cyclin D1* Promoter

Kaiso is a dual-specificity transcription factor with sequence- and methyl-CpG-specific transcriptional repression ability [10], [19], [21]. In this study we showed that Kaiso exhibits dual-specificity DNA binding to the human *cyclin D1* promoter; Kaiso bound to the -1067 KBS region of the *cyclin D1* promoter in a sequence-specific manner and it bound multiple CpG rich sites within the *cyclin D1* promoter region in a methylation-dependent but KBS-independent manner (Figures 1, 2, 3). While the significance of Kaiso’s sequence-specific versus methyl-CpG-specific DNA binding remains largely unknown, our data shows that both types of DNA-binding can occur independently at one gene promoter locus. Previously, Prokhortchouk *et* *al.,* [19] showed that the Kaiso zinc fingers preferentially associate with consecutive methylated CpG-dinucleotides, and that binding affinity decreases if there are one or more nucleotides between the consecutive CpG-dinucleotides. While our findings support those of Prokhortchouk *et al.*, we also showed that the presence of consecutive CpG-dinucleotides is not a strict requirement for Kaiso DNA binding (Figure 2A & B; CpG2 oligonucleotide). Furthermore, binding to methyl-CpG sites can also occur in the presence of a core KBS, as observed in this study.

The presence of a core KBS sequence in one of the CpG rich regions motivated us to examine Kaiso binding to this region using an oligonucleotide (CpG7) containing the KBS and CpGs. We found that Kaiso was able to bind this +69 core KBS region in a methyl-CpG-specific manner, and that binding required the presence of the two CpG-dinucleotides upstream of the core KBS (Figure 4). Mutation of this core KBS sequence decreased but did not abolish Kaiso binding, suggesting that the role of this core KBS in close proximity to single CpGs is most likely to stabilize Kaiso DNA binding. Our data support those of Sasai *et al.,* [7], who demonstrated that Kaiso and the Kaiso-like zinc finger protein ZBTB4 bind single methylated-CpG sites with higher affinity if a core KBS was present [7]. However, it is possible that high affinity Kaiso binding requires two consecutive methylated-CpG sites in the absence of a core KBS. While previous studies have implicated *cyclin D1* as a Kaiso target gene [10], [20], our study is the first to demonstrate Kaiso’s dual-specificity (sequence- and methylation-specific) DNA-binding and transcriptional repression of the *cyclin D1* promoter in mammalian cells.

Importantly, we confirmed that Kaiso also associated with the −1067, +69 core KBS and CpG regions of the *cyclin D1* promoter *in vivo* in both MCF7 breast and HCT 116 colon carcinoma cells (Figures 1, 2, 3). However, since the +69 KBS, CpG5 and CpG8 sites are in close proximity to each other within the promoter, it is possible that Kaiso associates with one and or all 3 sites simultaneously; however it will be difficult to resolve these sites *in vivo* using ChIP assays. Nevertheless, our data indicates that Kaiso’s binding to the *cyclin D1* promoter is not cell line specific and supports our hypothesis that *cyclin D1* is a *bona fide* Kaiso target gene. While we do not know if the Kaiso-*cyclin D1* promoter association is preserved in other cell types such as fibroblasts, we recognize the need to pursue such studies.

Our minimal promoter-reporter assays demonstrated that Kaiso overexpression resulted in dose-dependent repression of the *cyclin D1* promoter and further validated *cyclin D1* as a Kaiso target gene (Figure 5). This is consistent with the findings of Park *et al.* who previously reported that Kaiso was a negative regulator of *cyclin D1* expression in *Xenopus* [10], and the findings of Jiang *et al*. who recently demonstrated that Kaiso overexpression decreased cyclin D1 protein levels in lung cancer cells [20]. However, neither study determined Kaiso’s mechanism of transcriptional repression of the *cyclin D1* promoter. Here we demonstrate that Kaiso-mediated transcriptional repression of *cyclin D1* occurred in a KBS sequence-specific and methyl-CpG-dependent manner (Figures 5 & 6). Our findings suggest that the KBS and methyl-CpG-dinucleotides are physiologically relevant for Kaiso-mediated transcriptional repression of *cyclin D1*. Interestingly, when both CpG and KBS sites were inactivated (due to demethylation and KBS mutations respectively), Kaiso overexpression had minimal effect on the *cyclin D1* promoter-reporter activity (Figure 6D). Collectively, our findings suggest that Kaiso binds and negatively regulates the *cyclin* D1 minimal promoter via two distinct mechanisms that involve the sequence-specific KBS or the methyl-CpG sites. Since mammalian DNA methylation is an essential epigenetic modification associated with transcriptional repression, our findings implicate Kaiso in both sequence-specific and methylation-dependent gene regulation of the *cyclin D1* promoter.

Finally, the increased cell proliferation observed in the HCT 116 Kaiso-depleted cells supports our hypothesis that *cyclin D1* is a *bona fide* Kaiso target gene. Since the Wnt pathway is constitutively active in HCT 116 cells and many other factors regulate *cyclin D1* expression and function, it is not surprising that we only observed an ∼ 1.7-fold increase in cyclin D1 protein levels in HCT 116 depleted cells and a modest decrease in cyclin D1 protein levels upon Kaiso overexpression in MCF7 cells.

Whether Kaiso exhibits preferential binding to the KBS over the CpG sites in the *cyclin D1* promoter *in vivo* remains to be determined, and may be context dependent. Nevertheless, our data show a relationship between Kaiso and the cell cycle regulator *cyclin D1* in mammalian cells. Together our experiments demonstrate that the POZ-ZF transcription factor Kaiso associates with the *cyclin D1* promoter with dual-specificity and represses *cyclin D1* expression. However, the physiological relevance of this unique dual-specificity mechanism of transcriptional regulation of *cyclin D1* and other Kaiso target genes remains to be determined.

## Supporting Information

Figure S1 **GST-Kaiso fusion proteins.** 5 µg of purified GST-Kaiso fusion proteins utilized in EMSA studies were resolved on an SDS-PAGE gel to confirm expression and integrity of proteins.
(TIFF)

Figure S2 **Chromatin Immunoprecipitation negative control.** Primers designed to amplify a region located at +326 to +526 bp of the *cyclinD1* promoter (which lacked KBS sites) were used as a negative control to confirm the specificity of Kaiso binding to the −1067, +69 and CpG sites of the *cyclinD1* promoter in MCF7 cells.
(TIFF)

Figure S3 **Kaiso overexpression alters cyclinD1 expression in MCF7 cells.** (**A**) Transient transfection of MCF7 cells with the Kaiso expression vector (pcDNA3.1-hKaiso) resulted in an ∼ 1.7 fold decrease in cyclinD1 protein levels.
(TIFF)
