# Supplementary figures and images for: Kaiso Represses the Cell Cycle Gene cyclin D1 via Sequence-Specific and Methyl-CpG-Dependent Mechanisms
Source: PLoS One. 2012 Nov 30;7(11):e50398. doi: 10.1371/journal.pone.0050398 (PMC3511522; doi:10.1371/journal.pone.0050398)

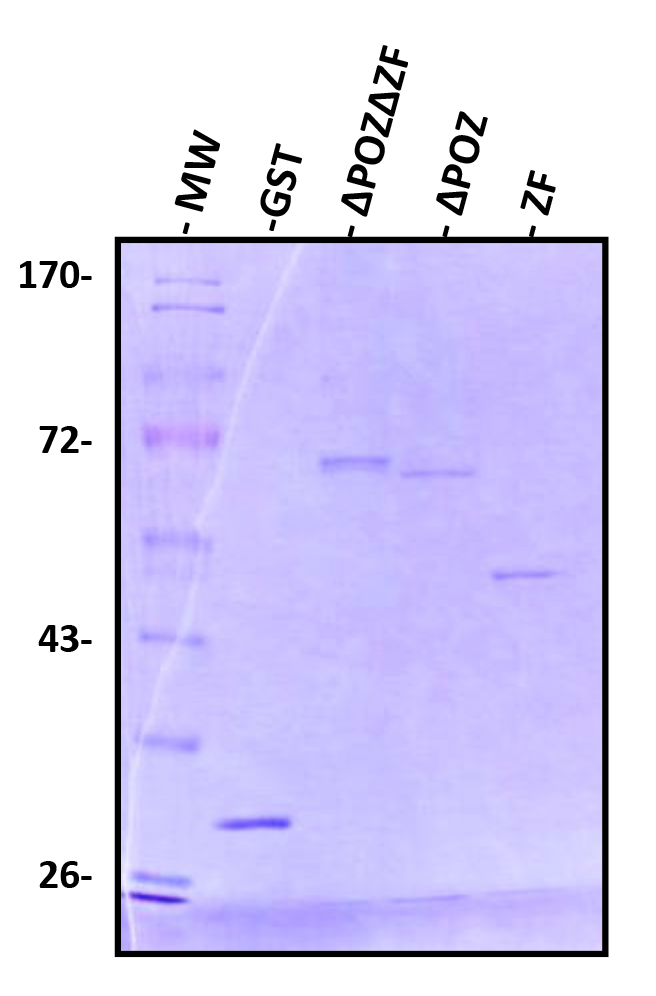

Supplement: Figure S1 — GST-Kaiso fusion proteins. 5 µg of purified GST-Kaiso fusion proteins utilized in EMSA studies were resolved on an SDS-PAGE gel to confirm expression and integrity of proteins. (TIFF) [file pone.0050398.s001.tiff]

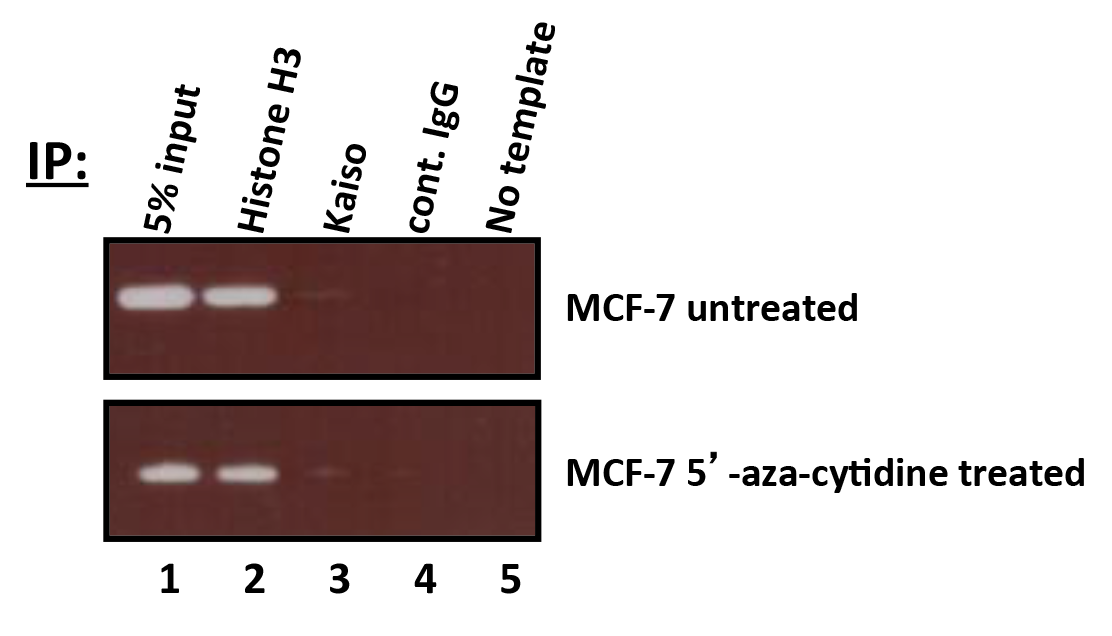

Supplement: Figure S2 — Chromatin Immunoprecipitation negative control. Primers designed to amplify a region located at +326 to +526 bp of the cyclinD1 promoter (which lacked KBS sites) were used as a negative control to confirm the specificity of Kaiso binding to the −1067, +69 and CpG sites of the cyclinD1 promoter in MCF7 cells. (TIFF) [file pone.0050398.s002.tiff]

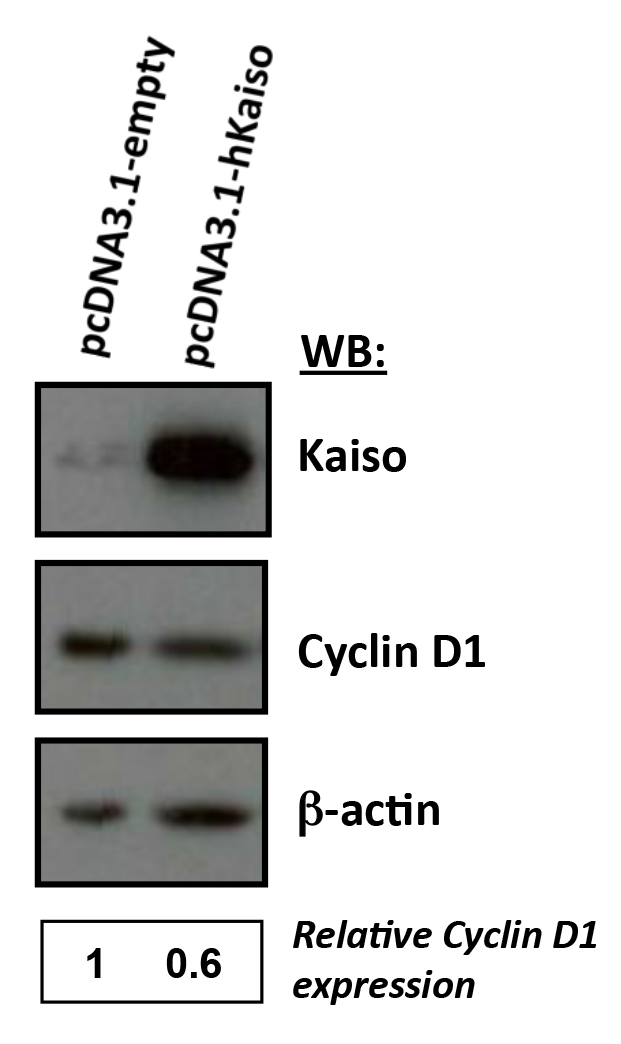

Supplement: Figure S3 — Kaiso overexpression alters cyclinD1 expression in MCF7 cells. (A) Transient transfection of MCF7 cells with the Kaiso expression vector (pcDNA3.1-hKaiso) resulted in an ∼ 1.7 fold decrease in cyclinD1 protein levels. (TIFF) [file pone.0050398.s003.tiff]
